# Echocardiographic assessment of mitral valve morphology after Percutaneous Transvenous Mitral Commissurotomy (PTMC)

**DOI:** 10.1186/1476-7120-5-48

**Published:** 2007-12-08

**Authors:** Hosam Hasan-Ali, Hamdy Shams-Eddin, Alaa A Abd-Elsayed, Muhammad H Maghraby

**Affiliations:** 1 Department of Cardiovascular Medicine, Assiut University Hospitals, Assiut, Egypt; 2 Department of Public Health and Biostatistics, Faculty of Medicine, Assiut University, Assiut, Egypt; 3 Department of Internal Medicine, Assiut University Hospitals, Assiut, Egypt

## Abstract

**Aims:**

PTMC produces significant changes in mitral valve morphology as improvement in leaflets mobility. The determinants of such improvement have not been assessed before.

**Methods and results:**

The study included 291 symptomatic patients with mitral stenosis undergoing PTMC. Post-PTMC subvalvular splitting area was a determinant of post-PTMC excursion in both the anterior (B 0.16, 95% CI 0.03 to 0.30, p < 0.05) and the posterior (B 0.12, 95% CI 0.01 to 0.24, p < 0.05) leaflets. Another determinant was the post-PTMC transmitral pressure gradient for anterior (B -0.02, 95% CI -0.04 to -0.005, p < 0.01) and posterior (B -0.01, 95% CI -0.04 to -0.005, p < 0.05) leaflets excursion. The relationship between post-PTMC MVA and leaflet excursion was non-linear "S curve". There was a steep increase of both anterior (p, 0.02) and posterior (p, 0.03) leaflets excursion with increased MVA till the MVA reached a value of about 1.5 cm^2^; after which both linear and S curves became nearly parallel.

**Conclusion:**

The improvement in leaflets excursion after PTMC is determined by several morphologic and hemodynamic changes produced in the valve. The increase in MVA improves mobility within limit; after which any further increase in MVA is not associated by a significant improvement in mobility in both leaflets.

## Background

Percutaneous transvenous mitral commissurotomy (PTMC) was found to be associated with splitting of the fused mitral commissures with a subsequent increase in the mitral valve area (MVA) [1-7]. However, not all patients with commissural splitting after the procedure were found to have an optimal MVA [6]. This suggested that the mechanism of successful PTMC may be more complex than was reported previously. Short term improvements in MVA and symptoms that occur when commissures are not split may be attributed to other mechanisms, such as improvement of leaflet mobility secondary to disruption of the diseased submitral tissue [7]. The historical obsession of the MVA limited more creative ways of looking at the mitral valve. Assessment of the other changes produced in mitral valve morphology may have an adjuvant value to the conventional measurement of the MVA in the morphologic assessment of the mitral valve function. Only few reports referred to the improvement in valve mobility [8,9]. The determinants of the extent of such improvement have not been assessed before. This study aimed to determine the changes produced in mitral valve morphology and to test the determinants of improved leaflet excursion after PTMC.

## Methods

The study included symptomatic patients with mitral stenosis with MVA ≤ 1.5 cm^2^, Wilkins' score ≤ 10, isolated mitral stenosis or with ≤ grade II mitral regurgitation, and giving an informed consent to the procedure. Patients with poor echocardiographic window, left atrial thrombus, interatrial septum thickness > 4 mm, recent thromboembolic event of less than 3 months duration, associated other valve lesions that need surgical correction, and associated significant coronary artery disease were excluded. The study was approved by our faculty ethical committee and was adherent to the regulations of the declaration of Helsinki.

### Echocardiographic studies

Transthoracic echocardiography (TTE) was done the day before PTMC and 24–48 hours after (before discharge), using real time ultrasound imaging system (ATL HDI-5000) with a phased array multifrequency 2–4 MHz transducer. The standard echocardiographic measurements were done and averaged in 4 cardiac cycles. These measurements were taken while the patient was on supine and left lateral decubitus positions. Preprocedural Wilkins' score was evaluated [10]. In addition special measurements were taken. These measurements were:

#### Valve mobility

**Figure 1:**
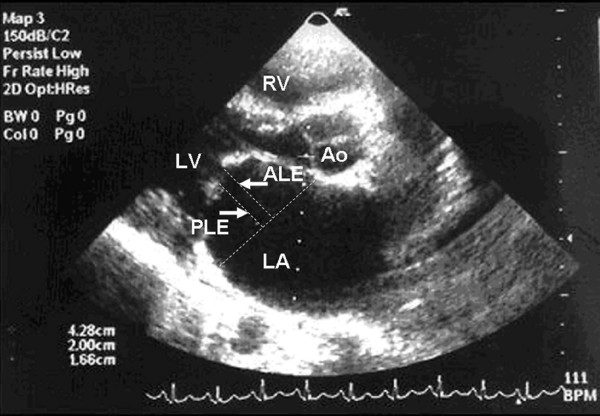
Anterior and posterior leaflets excursion measured in parasternal long axis view. RV, right ventricle; LV, left ventricle; LA, left atrium; Ao, aorta; ALE, anterior leaflet excursion; PLE, posterior leaflet excursion.

It was assessed by measuring the anterior and posterior leaflets excursion [8]. To measure the excursion; in the parasternal long axis view, at maximum doming in early diastole, a line was drawn at the level of the mitral valve annulus (at the base of the leaflets), and then 2 perpendicular lines were dragged from the tips of the leaflets on that line (Figure 1).

#### Subvalvular splitting

**Figure 2:**
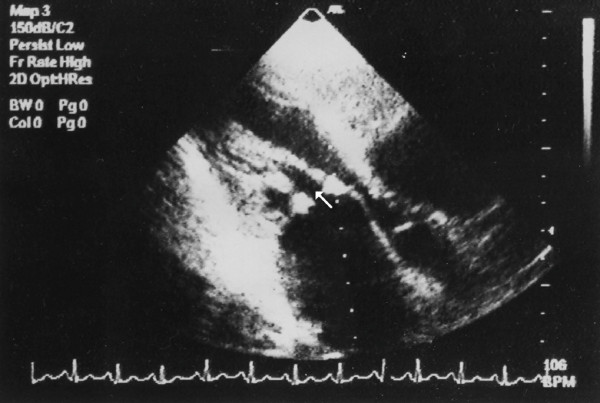
Subvalvular splitting area (white arrow) measured in apical long axis view.

It was assessed by measuring the area subtended by the papillary muscles and chordae tendinae below the mitral valve, measured at maximum doming of the valve leaflets in early diastole in apical long axis view; where the 2 papillary muscles and the chordae could be seen separated in profile (Figure 2).

#### Degree of commissural splitting

**Figure 3:**
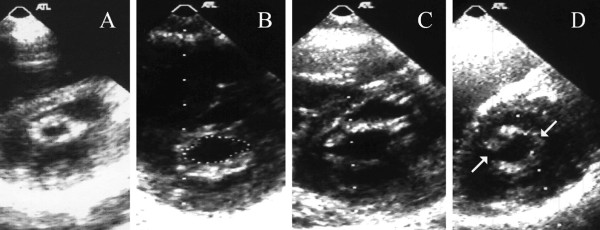
A. Bilateral commissural fusion with commissural score 0 in both commissures, B. Partial splitting of both commissures with score 0.5 in each (total score 1), C. Complete splitting of both commissures with score 1 in each (total score 2), D. Complete splitting of the posterior commissure with score 1 and partial splitting of the anterior commissure with score 0.5 (total score 1.5).

It was assessed in short axis parasternal view as regards the depth score of the splitted commissure. The score was assessed in each commissure as follows (No commissural splitting = 0, partial splitting of the commissure = 0.5, complete splitting of the commissure = 1); then the score of both commissures is summed to give a total score of commissural splitting. (Figure 3) This score is based on a nearly similar score used by Fernandez-Ortiz et al [11]. There score differs from ours in that it dealt with commissural splitting as a whole without predilection to the exact commissure splitted and gave a score of 0 to absent splitting, 1 to either partial or complete splitting of one commissure, score 1.5 to partial splitting of one commissure and complete splitting of the other and score 2 to complete splitting of both commissures.

#### Commissural calcium

It was analyzed in parasternal short axis view by careful scanning of the mitral valve orifice from the level of the aortic root to the mitral valve leaflets. The echogenesity of the aortic root was used as a point of reference [9]. The severity of commissural calcium was assessed in each commissure as follows (when absent = 0 and when present = 1); then the score of both commissures was summed to give a total score.

### Valvuloplasty Procedure

PTMC was performed using either the metallic valvotome [12] (67), the Inoue balloon [13] (107), the double balloon [14] (69) and multitrack [15] (48) techniques.

### Statistical analysis

Clinical and echocardiographic variables were prospectively collected to be analyzed using SPSS (Statistical Program for Social Sciences version 11 for windows, 2001, SPSS Inc., Chicago, IL, USA). Continuous variables were presented as a mean ± SD. Variables, before and after the procedure, were compared using paired student's t-test for continuous variables and Wilcoxon test for non-parametric data. Linear regression analysis was used to study the predictors of post-PTMC leaflets excursion. Variables failed to represent a linear relationship were tested using S-curve analysis. Values were considered significant with a p Value <0.05. Interobserver variability was assessed for the special echocardiographic measurements in 40 patients; 20 before and 20 after PTMC, by two independent echocardiographers (first 2 authors). Kappa statistics were used as a measure of agreement for categorical variables. Bland-Altman method was used to assess the interobserver variability for continuous variables (Prism 5 for windows statistical software). The bias (paired mean difference between the 2 echocardiographers) and 95% limits of agreement (2 SD around the mean difference) relative to the mean measurement of both echocardiographers were assessed.

## Results

**Figure 4:**
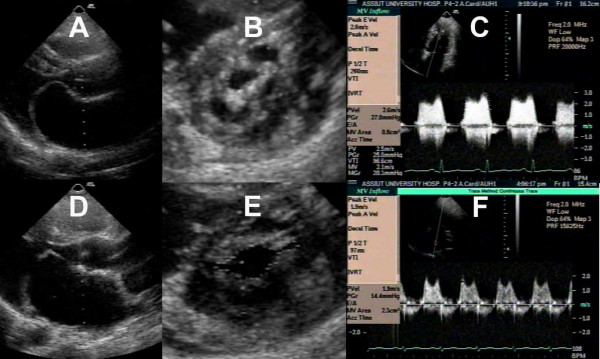
A case of mitral stenosis before and after PTMC in the parasternal long axis view (A, D), short axis view (B, E), and continuous wave Doppler transmitral flow (C, F).

The patient group aged 30 ± 9 years with Wilkins' score 7.0 ± 1.7. The patients' baseline characteristics are shown in table 1. PTMC produced significant changes in the mitral valve morphologic and hemodynamic characteristics (table 2.). PTMC was found to produce a significant increase in the MVA, splitting of the fused anterior and posterior commissures, and improvement in leaflet mobility; reflected as increased excursion of both leaflets (figure 4, see additional file 1). This was associated with a decrease in the transmitral pressure gradient, left atrial diameter, left ventricular end-diastolic diameter, and a significant increase in the left ventricular end-systolic diameter and ejection fraction.

**Table 1:** Patients' baseline characteristics

Age (years)	30 ± 9 (12–56)
Male gender	97(33%)
Patients' complaint:	
• Dyspnea	234 (80%)
• Low cardiac output	123 (42%)
• Palpitation	158 (54%)
• Previous systemic	3 (1%)
• embolism	83 (29%)
NYHA class ≥ III	131 (45%)
Atrial fibrillation	
Pulmonary hypertension:	13 (5%)
• No (<30 mmHg)	131 (40%)
• Mild (30–49 mmHg)	93 (32%)
• Moderate (50–69 mmHg)	67 (23%)
• Severe ≥ (70 mmHg)	
Wilkins' score	1.9 ± 0.5
• Mitral valve mobility	2.1 ± 0.5
• Mitral valve thickness	1.2 ± 0.9
• Mitral valve calcification	1.8 ± 0.6
• Subvalvular thickening	7.0 ± 1.7
• Total Wilkins' score	
Commissural calcification	198 (68%)
• No	65 (22%)
• Unicommissural	28 (10%)
• Bicommissural	

**Table 2:** Morphologic and hemodynamic changes in the mitral valve after PTMC

	Before PTMC	After PTMC	p Value
Anterior leaflet excursion (cm)	1.8 ± 0.5	2.1 ± 0.5	<0.0001
Posterior leaflet excursion (cm)	1.4 ± 0.4	1.6 ± 0.5	<0.0001
Subvalvular splitting area (cm^2^)	0.9 ± 0.3	1.4 ± 0.4	<0.0001
Anterior commissural splitting score:			<0.0001
- No splitting (0)	286 (98.3%)	10 (3.4%)	
- Partial splitting (0.5)	5 (1.7%)	80 (27.5%)	
- Complete splitting (1)	0	201 (69.1%)	
Posterior commissural splitting score:			<0.0001
- No splitting (0)	272 (93.5%)	21 (7.2%)	
- Partial splitting (0.5)	19 (6.5%)	86 (29.6)	
- Complete splitting (1)	0	184 (63.2%)	
Total commissural splitting score:			<0.0001
- 0	269 (92.4%)	0	
- 0.5	20 (6.9%)	12 (4.1%)	
- 1	2 (0.7%)	36 (12.4%)	
- 1.5	0	112 (38.5%)	
- 2	0	131 (45%)	
Mitral valve annular diameter (cm)	3.7 ± 0.6	3.6 ± 0.6	NS
Mitral valve area (cm^2^)	0.9 ± 0.2	2.0 ± 0.4	<0.0001
Transmitral pressure gradient (mmHg)	17 ± 7	5 ± 3	<0.0001
Left atrial diameter (cm)	5.2 ± 0.9	4.5 ± 0.8	<0.0001
Left ventricle end-diastolic diameter (cm)	4.3 ± 0.6	4.4 ± 0.6	<0.0001
Left ventricle end-systolic diameter (cm)	3.0 ± 0.5	2.9 ± 0.5	<0.0001
Ejection fraction (%)	65 ± 10	70 ± 8	<0.0001

PTMC, percutaneous transvenous mitral commissurotomy; NS, not significant

### Determinants of improved leaflet excursion after PTMC

**Figure 5:**
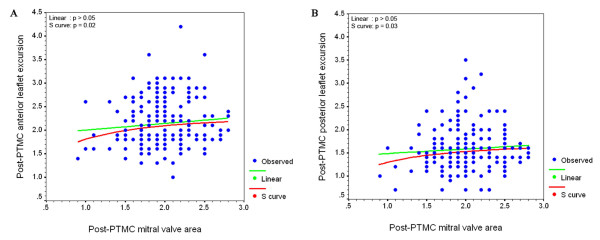
Relationship between post-PTMC mitral valve area and leaflet excursion.

Linear regression analysis (table 3) showed that post-PTMC subvalvular splitting area was a weak univariate determinant of post-PTMC excursion in both the anterior (B 0.16, 95% CI 0.03–0.30, p < 0.05) and the posterior (B 0.12, 95% CI 0.01–0.24, p < 0.05) leaflets. Also, post-PTMC transmitral pressure gradient was a significant determinant of anterior (B -0.02, 95% CI -0.04 to -0.005, p < 0.01) and posterior (B -0.01, 95% CI -0.04 to -0.005, p < 0.05) leaflets excursion. Preprocedural Wilkins' score and its 4 parameters, commissural calcium, post-PTMC MVA, commissural splitting score, left atrial diameter, left ventricle dimensions, ejection fraction, and degree of mitral regurgitation failed to predict it. We found that the relationship between post-PTMC MVA and leaflet excursion was non-linear "S curve" as shown in figure 5. There was a steep increase of both anterior and posterior leaflets excursion with increased MVA till the area reached a value of about 1.5 cm^2^; after which both linear and S curves became nearly parallel.

**Table 3:** Linear predictors of improved leaflet excursion after PTMC

	Anterior leaflet excursion B (95% CI)	Posterior leaflet excursion B (95% CI)
Age	-0.001 (-0.006 to 0.008)	-0.002 (-0.008 to 0.005)
Effective dilating diameter	0.007 (-0.003 to 0.017)	0.01 (-0.001 to 0.018)
Valve mobility	-0.02 (-0.08 to 0.04)	-0.03 (-0.08 to 0.08)
Valvular thickening	-0.01 (-0.04 to 0.08)	-0.04 (-0.06 to 0.08)
Valvular calcification	-0.01 (-0.06 to 0.07)	-0.02 (-0.07 to 0.04)
Subvalvular thickening	-0.03 (-0.09 to 0.05)	-0.05 (-0.09 to 0.08)
Wilkins' score	-0.01 (-0.04 to 0.04)	-0.06 (-0.08 to 0.04)
Commissural calcium score	-0.02 (-0.07 to 0.01)	-0.03 (-0.11 to 0.05)
Mitral valve area	0.12 (-0.03 to 0.31)	0.10 (-0.05 to 0.15)
Subvalvular splitting area	0.16 (0.09 to 0.20)*	0.12 (0.06 to 0.20)*
Anterior commissural splitting score	0.14 (-0.03 to 0.18)	0.08 (-0.02 to 0.11)
Posterior commissural splitting score	0.01 (-0.02 to 0.06)	0.07 (-0.10 to 0.11)
Total commissural splitting score	0.08 (-0.03 to 0.1)	0.002 (-0.002 to 0.008)
Left atrial diameter	0.05 (-0.04 to 0.11)	0.05 (-0.02 to 0.09)
Left ventricle end-diastolic diameter	0.06 (-0.04 to 0.1)	0.05 (-0.05 to 0.1)
Left ventricle end-systolic diameter	0.04 (-0.08 to 0.11)	0.02 (-0.03 to 0.1)
Ejection fraction	0.001 (-0.007 to 0.008)	0.001 (-0.005 to 0.008)
Transmitral pressure gradient	-0.02 (-0.04 to -0.005)†	-0.01 (-0.04 to -0.005)*
Degree of mitral regurgitation	-0.005 (-0.09 to 0.002)	-0.006 (-0.09 to -0.001)

CI, confidence interval. * p < 0.05, † p < 0.01

### Interobserver variability

**Figure 6:**
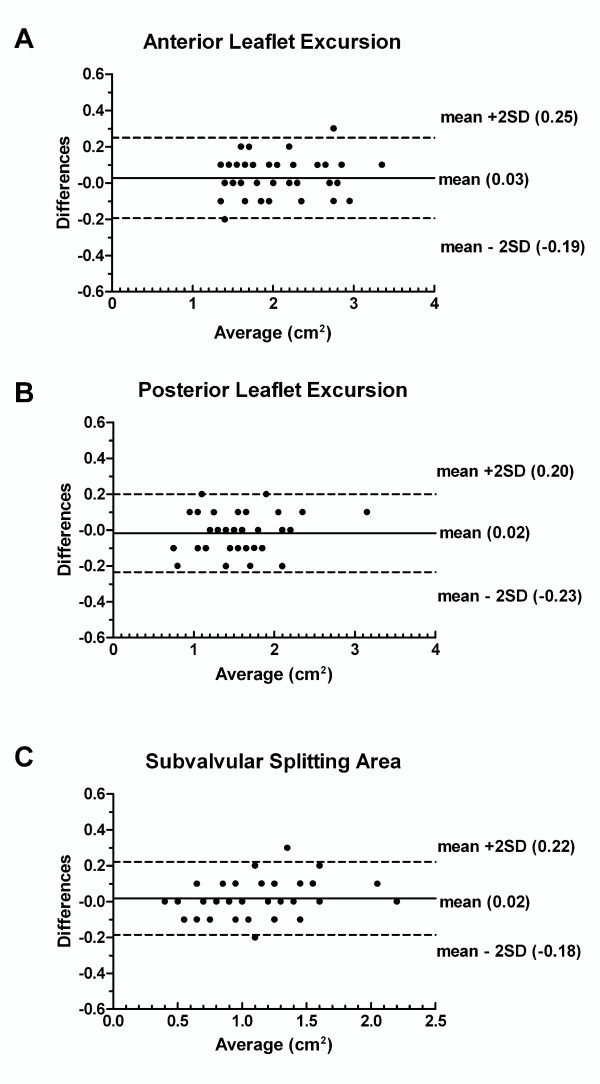
Bland-Altman plots for interobserver variability in anterior leaflet excursion (A), posterior leaflet excursion (B), and subvalvular splitting area (C).

The measurements taken by the two echocardiographers were reproducible for commissural calcium score (Kappa = 0.9, approximate significance <0.0001), anterior commissural splitting score (Kappa = 0.87, approximate significance <0.0001), posterior commissural splitting score (Kappa = 0.88, approximate significance <0.0001), total commissural splitting score (Kappa = 0.85, approximate significance <0.0001). Measurements of leaflets excursion and subvalvular splitting area were also found to be reproducible as shown in figure 6.

## Discussion

Over the past several years PTMC has become an accepted alternative to surgery in the treatment of patients with mitral stenosis.3–4 previous studies have confirmed that this procedure is highly successful with a low complication rate and significant short- and long- term improvement in both hemodynamics and patient symptoms [3,4,16].

The mitral valve is a complex structure consisting of 6 components; annulus fibrosus, leaflets, chordae tendinae, papillary muscles, left atrial wall, left ventricular wall. These 6 components should be considered functionally as a unit, since derangement of any one part may allow serious hemodynamic consequences [17]. The present study demonstrates that PTMC produces significant morphologic and hemodynamic changes in the mitral valve.

In accordance of the published data [1,2,11], we found that PTMC produced a significant increase in MVA and a significant splitting of both mitral commissures. It was reported in the early pathologic studies that subvalvular fibrosis with fusion and shortening of the chordae tendinae causes obliteration of the interchordal spaces which are considered as secondary orifices for blood flow, below the main orifice formed by the leaflets. Commissural fusion causes valvular stenosis and is affected in 76% of cases. Subvalvular fibrosis causes a second level of obstruction to blood flow "subvalvular" and is present in 39% of cases of MS [18]. It was, also, reported that in mitral stenosis the chordae are occasionally retracted that the leaflets appear to be inserted directly into the papillary muscles, so that the interchordal spaces are entirely obliterated. They recommended that mitral commissurotomy on such a valve must include splitting of the papillary muscles as well as the commissures [19]. It was suggested that, unlike open mitral commissurotomy (during which subvalvular fusion can be directly visualized and chordae tendinae and papillary muscles can be manually separated), PTMC more closely resembles closed mitral commissurotomy, which has a limited effect on subvalvular apparatus [9]. Nevertheless; the effect of PTMC on the subvalvular apparatus was not well studied. This is properly because echocardiography has been considered to have a limited ability in visualizing the chordae tendinae. In rheumatic heart disease, thickening, fusion, and shortening of the chordae could facilitate studying the subvalvular structures; either qualitatively as a part of a scoring system for mitral valve morphology [9,10], or qualitatively by measuring the subvalvular distance ratio transthoracically [20]. Previous reports limited echocardiographic assessment of subvalvular structures preprocedurally to predict rather to detect successful outcome. In our study, we used the subvalvular splitting area as a marker of subvalvular fusion assessed in a quantitative way before and after the procedure. We found this area increased significantly after PTMC. Similar to the published reports from the eastern countries endemic in rheumatic heart disease [5,20], the patients in our study were young. This properly provided a good image quality to take subvalvular splitting measurement. It is not known if such measurements could be easily applicable in older patients seen in western countries.

PTMC also caused a significant improvement in valve mobility, reflected as increase in both anterior and posterior leaflets excursion. Leaflet excursion was found to increase with increased post-PTMC MVA till the MVA reaches a value around 1.5 cm^2 ^after which the relationship became non-significant. Also, excursion was determined by post-procedural subvalvular splitting area, and transmitral pressure gradient. It was supposed that the valve mobility is the result of all the pathologic processes in the mitral valve apparatus that results in stenosis rather than an independent variable. So, it is affected by the degree of commissural fusion, leaflet thickness, calcification and subvalvular fusion [21]. Commissural splitting failed to predict the post procedural leaflet excursion. This is possibly because the method used for quantification of commissural splitting is too crude to be correlated with the fine changes in leaflet excursion. It is well known from the previous in vitro studies that the main mechanism of increase in the MVA after PTMC is splitting of the fused mitral commissures [1,2]. So, measurement of MVA by planimetry can be considered as an indirect fine quantitative measure of commissural splitting, and according to our findings we can say that, the improvement in valve mobility has a maximum limit, regardless of the extent of commissural splitting produced and the increase in MVA. This limit is probably defined by the degree of valve pathology. According to our data, leaflets excursion increase significantly with increased MVA till the area reaches a value of about 1.5 cm^2^, after which any further increase in MVA was not associated with any further increase in leaflet excursion in both leaflets. It is to be noted that a MVA of 1.5 cm^2 ^is used in the literature as a cut off value to define an optimal outcome after PTMC [12,14-16].

## Conclusion

PTMC is associated with significant changes in mitral valve morphology in terms of splitting of the fused mitral commissures, increased MVA, improved leaflet excursion, and splitting of the subvalvular structures. The improvement in leaflet excursion after PTMC is determined by several morphologic and hemodynamic changes produced in the valve. The increase in MVA improves leaflet mobility within limit; till a value area around 1.5 cm^2^, after which any further increase in MVA is not associated by a significant improvement in mobility in both the anterior and posterior leaflets.

## Competing interests

The author(s) declare that they have no competing interests.

## Authors' contributions

HH-A participated in the study conception and design, valvuloplasty procedure, acquisition of clinical and echocardiographic data, data analysis and interpretation, and wrote the manuscript. HS-E participated in valvuloplasty procedure, acquisition of clinical and echocardiographic data, and critical manuscript revision. AAA-E participated in the study design, data analysis, drafting of the manuscript, critical manuscript revision and gave important suggestions. MHM participated in clinical support, coordination and gave important contributions in critical manuscript revision.

All authors have read and approved the final manuscript.

## Supplementary Material

Additional File 1 An echocardiographic case presentation. This file represents the echocardiographic assessment of the mitral valve morphology of a 22 years old female patient before and after percutaneous transvenous mitral commissurotomy. Click here for file
